# Reversal of transforming growth factor-β induced epithelial-to-mesenchymal transition and the ZEB proteins

**DOI:** 10.1186/1755-1536-5-S1-S28

**Published:** 2012-06-06

**Authors:** Shreyasi Das, Bryan N Becker, F Michael Hoffmann, Janet E Mertz

**Affiliations:** 1 Sanford-Burnham Medical Research Institute, 10901 N. Torrey Pines Road, La Jolla, CA 92037, USA; 2 Department of Medicine, University of Wisconsin School of Medicine and Public Health, 600 Highland Avenue, Madison, Wisconsin 53792, USA; 3 Laboratory of Genetics, University of Wisconsin School of Medicine and Public Health, 425-G Henry Mall, Madison, Wisconsin 53706, USA; 4 McArdle Laboratory for Cancer Research, University of Wisconsin School of Medicine and Public Health, 1400 University Ave, Madison, Wisconsin 53706, USA

## Abstract

**Background:**

The dynamic process of epithelial-to-mesenchymal transition (EMT) is a causal event in kidney fibrosis. This cellular phenotypic transition involves activation of transcriptional responses and remodeling of cellular structures to change cellular function. The molecular mechanisms that directly contribute to the re-establishment of the epithelial phenotype are poorly understood.

**Results:**

Here, we discuss recent studies from our group and other laboratories identifying signaling pathways leading to the reversal of EMT in fibrotic models. We also present evidence that transcriptional factors such as the ZEB proteins are important regulators for reversal of EMT.

**Conclusion:**

These studies provide insights into cellular plasticity and possible targets for therapeutic intervention.

## Background

Kidney failure is a serious medical problem with limited therapeutic choices available. The kidney is a vital organ in the body as it regulates blood pressure, volume, pH, and levels of electrolytes and metabolites [1]. Proximal tubule epithelial cells are one of the key components of the kidney as they carry out the cellular functions necessary for filtering the blood. Epithelial cells can loose filtering functions during injury to the kidney when exposed to cytokines such as transforming growth factor β (TGF-β) [2]. In many cases, injury to the kidney is temporary, with epithelial cells able to regenerate. However, when diseases such as diabetes, hypertension, and polycystic kidney disease are poorly controlled, chronic injury to the proximal tubule epithelial cells, and eventually, kidney failure can occur. Unfortunately, doctors and patients can only manage the medical problems that present themselves following kidney damage since there are no treatments currently available to regain kidney function. Thus, understanding the molecular mechanisms leading to kidney disease and developing specific therapies is of great importance.

### Basics of epithelial-to-mesenchymal transition as a biological process

Epithelial-to-mesenchymal transition (EMT) is the major causative event of kidney cells loosing function during injury of the kidney and chronic kidney disease [3,4]. The principle behind EMT is polarized epithelial cells in well organized layers convert to individual, motile fibroblasts [4-7]. The transition is characterized by the loss of epithelial morphology, reduction of cell-cell contacts, transcriptional repression of E-cadherin expression, degradation of cell-matrix adhesions, and rearrangement of the actin cytoskeleton. The mesenchymal fibroblasts in culture appear as long, spindle-shaped cells, capable of migration and possessing the ability to produce extracellular matrix. The changes that occur during the course of EMT do not only affect the cell itself, but also the environment surrounding the cell [8-10]. This process is reversible in normal processes such as gastrulation and organogenesis of the heart, musculoskeletal system, and the peripheral nervous system as well as in diseased states such as primary tumor metastasis [3,6]. Thus, one way to regain kidney function would be to find therapeutic agents that can reverse EMT.

### Transforming growth factor-β is an essential inducer of EMT

When basement membranes are damaged, the attached epithelial cells secrete cytokines to initiate EMT [3,8,10]. Growth factors such as transforming growth factor-β (TGF-β) are required to induce and maintain EMT; they do so via both Smad-dependent and -independent signaling events [7-15]. Several *in vivo *and *in vitro *models of fibrosis exhibit increased TGF-β1 expression in damaged tissues [3,6,10]. The cellular signaling commences by TGF-β ligand binding to a heteromeric complex of transmembrane serine/threonine kinases, type I and type II receptors (TβRI and TβRII) [15]. TβRII transphosphorylates TβRI, activating its kinase function. Functioning as a docking site, TβRI then directly phosphorylates Smad2 and Smad3 at carboxy-terminal serines [16,17]. Phosphorylated Smad2/3 associates with Smad4 in the cytoplasm. This complex is then translocated to the nucleus where it accumulates [17]. The Smad complex sequence-specifically binds to sites in the DNA, interacting with transcriptional coactivators or corepressors to regulate gene expression. Numerous studies have demonstrated that the TGF-β target genes regulating EMT are controlled through Smad signaling [7,13,14]. Over-expression of Smad 2 or Smad3 leads to an increase in EMT in mammary epithelial cells. Smad3 knockout mice exhibit amelioration of epithelial degeneration as demonstrated in a lens injury model. SB431542, a potent inhibitor of TGF-β Receptor Type I (TβRI) kinase activity blocks TGF-β-induced EMT in Namru Murine Mammary gland (NMuMG) cells [18,19]. Over-expression of mutant TRβI that lacks the ability to bind to Smad2/3 inhibits TGF-β-induced EMT in NMuMg cells [20]. Over-expression of inhibitory Smad7 blocks fibrosis in renal UUO models and EMT in retinal epithelium *in vitro *[21]. Lastly, Zavadil and colleagues demonstrated that TGF-β fails to fully induce EMT morphology and to stimulate key transcriptional regulators in primary murine proximal tubules cells isolated from Smad3 knockout kidneys [22].

### Macromolecules may be useful to understand which pathways are important for EMT reversal

In renal fibrosis, tubular epithelial cells undergo EMT in response to insults generated by diseases such as diabetes or hypertension [23,24]. The converted fibroblasts produce excessive extracellular matrix, eventually resulting in end-state organ failure where there is currently no method of curing available [8]. Most studies concentrate only on blocking EMT; rarely has a study actually reversed EMT.

Bone morphogenic protein-7 (BMP-7) is a member of the TGF-β superfamily [15,25,26]. Its recombinant protein ligands are in clinical trials as therapeutics for healing bone fractures [27]. Currently, BMP-7 is the most successful pre-clinical candidate to reverse EMT [2,24,28]. TGF-β1-induced EMT was reversed by treatment for 48 hours with 100 pM BMP-7 as observed by re-establishment of E-cadherin at cell junctions. BMP-7 can reverse fibrosis in an adult kidney. BMP-7 was administered in a chronic injury mouse model and produced amelioration of kidney fibrosis after one week. The EMT reversal was accomplished by BMP-7 antagonizing the TGF-β pathway and activating the epithelial transcriptional programming through Smads 1, 5, and 7 [28,29]. However, it remains to be elucidated which epithelial and mesenchymal genes are regulated by BMP-7.

Hepatocyte growth factor (HGF) is another macromolecule that has been studied for its ability to reverse EMT by antagonizing the TGF-β pathway, doing so through induction of expression of the Smad-binding inhibitory protein SnoN [30]. Administration of HGF partially reverses TGF-β-induced EMT in mouse kidney cells [31,32]. Unlike BMP-7, which is a potent reversal agent of EMT, HGF fails to completely reverse EMT in an *in vivo *UUO mouse model; a fibrotic marker is still observed in the cells [33]. This finding can be explained by HGF acting as an inducer of an EMT-like phenotype termed reversible scatter, suggesting that HGF has dual functions during cellular differentiation [34].

## Results and discussion

### Small molecules targeting cellular signaling protein intermediates both elucidate major EMT signaling events and can be developed as agents to reverse EMT

The studies using macromolecules such as BMP and HGF elucidated major signaling pathways important for EMT reversal. However, specific signaling pathway intermediates and transcription factors that maintain the mesenchymal program were still unknown. To identify factors that maintain the mesenchymal state, our group tested five different kinase inhibitors targeting TβRI, p38 mitogen-activated protein kinase (p38 MAPK), MAP kinase kinase/extracellular signal-regulated kinase activator kinase (MEK1), c-Jun NH-terminal kinase (JNK), and Rho kinase (ROCK) with SB431542, SB203580, U0126, SP600125, and Y27632, respectively, for their ability to reverse EMT induced by TGF-β1 in primary renal cells isolated from a TGF-β1 knockout mouse (mTEC-KO) [19,35-40]. The mTEC-KO cells were initially exposed to TGF-β1 to induce EMT, the kinase inhibitor was added, and the cells were examined for signs of reversal to an epithelial state [40]. Treatment with the single kinase inhibitors listed above did not completely reverse the mesenchymal phenotype as depicted by gene expression and morphological changes. Of the five individual inhibitors, only the TβRI inhibitor, SB431542, partially ameliorated the EMT phenotype by aiding in the reappearance of epithelial cadherins and decreasing mesenchymal gene expression. These findings suggested that blocking the TGF-β signaling pathway during the mesenchymal state only regulates gene expression, but does not induce the structural changes required to re-establish the epithelial phenotype.

Since EMT effects are mediated by multiple cellular pathways, a combination of the kinase inhibitors was used to understand if two intracellular signals need to be blocked for EMT reversal to occur. This idea is consistent with a study that reported reversal of a mesenchymal phenotype caused by conditional Fos over-expression in EpH4 through the use of a combination of constitutively active E-cadherin and a small molecule inhibitor of TGF-β, BIBU 3029 [41]. When our TGF-β-induced mesenchymal cells were incubated for 24 hours with the TβRI inhibitor SB431542 in combination with either the p38 MAPK inhibitor SB203580 or the RhoA inhibitor Y27632, the epithelial appearance of the cells was restored as indicated by a reduction in stress fibers and mesenchymal gene expression [40]. Taken together, these findings indicate that TGF-β maintains the mesenchymal phenotype through sustained activation of Smad-dependent transcriptional responses and elements downstream of ROCK or p38 MAPK.

The use of small molecule inhibitors of individual protein kinases not only demonstrates their potential for dissecting mechanisms of signal transduction for specific ligands and for delineating their roles in biologic responses, but also their potential as therapeutic agents. However, it remains to be seen which molecules will be useful for EMT reversal in clinical models.

### The ZEB protein family is an attractive target as an EMT reversal agent

The loss of transcriptional repressors from the promoter regions of genes central to the epithelial phenotype is a possible mechanism for the re-expression of these epithelial-specific genes during the reversal of EMT induced by macromolecules or cellular signaling kinase inhibitors. Smad-dependent signaling up-regulates expression of several transcription factors important for EMT induction, including Snail (Snai1), Slug (Snai2), Twist, and members of the ZFH family, ZEB1 (also called EF1, TCF8, AREB6, ZFHEP, NIL-2A, ZFHX1A, and BZP) and ZEB2 (also called SIP1, SMADIP1, ZFHX1B, and KIAA0569), making them good candidates to regulate the reversal of EMT [42-44]. These transcription factors activate EMT by binding to elements present in the promoter regions of a number of epithelial-specific genes including the E-cadherin promoter, thus suppressing synthesis of this cell-cell adhesion protein [7,45-47]. The involvement of the ZEB transcription factors is particularly tantalizing as an EMT reversal target since they regulate gene expression critical for both organ development and cancer metastasis [48]. The loss of ZEB2 in fetal mice results in a number of developmental defects, including the loss of migratory capabilities of neural crest cells [49]. Others have provided evidence for the role of the ZEB proteins during the induction of EMT by their repressing expression of E-cadherin and other epithelial structural components necessary for epithelial phenotype [44,50,51]. Additionally, the microRNA 200 family induces mensenchymal-to-epithelial transition in certain cancer cell lines [52]. Mutations in the *TCF8 *gene (GenBank accession number NM 030751) result in a mesenchymal-to-epithelial transition (MET) in mouse embryos by reprogramming gene expression, leading to developmental defects by diminishing progenitor cell proliferation and cell migration [53]. In Madin-Darby Canine Kidney (MDCK) cells, EMT is preceded by the loss of mature microRNA200a-c, inducing up-regulation of ZEB1 and ZEB2 expression followed by loss of E-cadherin expression and transition to the mesenchymal state. Taken together, these studies indicate transcriptional factors such as the ZEB proteins may be a more specific target for fibrotic therapeutics than drugs that interfere with signaling pathway intermediates.

**Figure 1:**
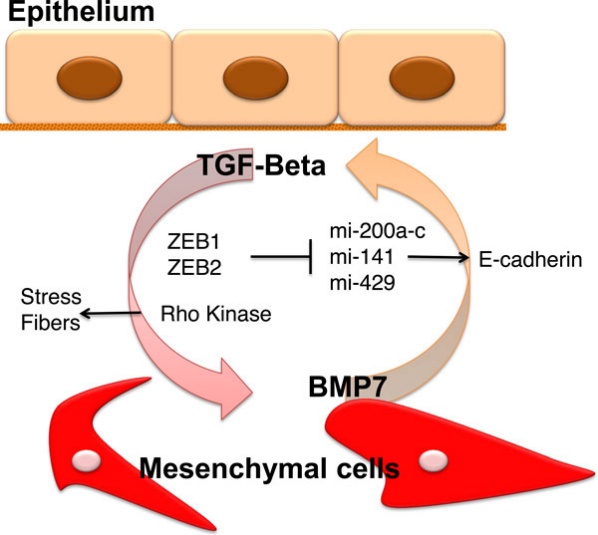
**Diagram of key events during EMT and MET**. TGF-β induces five events for epithelial cells to transition into the mesenchymal state: (i) loss of cell-cell contact, (ii) synthesis of mesenchymal proteins, (iii) re-arrangement of cell structural proteins such as actin, (iv) loss of basement membrane, (iv) loss of apical-basal polarity, and (v) the reverse, mesenchymal to epithelial transition can be induced by cytokines such as BMP-7.

Our group also examined the effects of ZEB1 and ZEB2 levels during the reversal of EMT Their expression is regulated by TGF-β [13], and they are highly expressed in fetal kidney cells [54]. NMuMG cells, a traditional EMT model [55] where ZEB is highly expressed [44], were incubated with TGF-β1 to induce EMT, and then the five kinase inhibitors targeting TβRI, p38 MAPK, MEK1, JNK, and ROCK were individually added as a reversal agent [40]. We observed that reversal of EMT by the TβRI inhibitor SB431542 involves, in part, inhibiting expression of ZEB1. Further studies demonstrated that depleting mesenchymal cells of ZEB1 and ZEB2 with specific shRNAs was insufficient to restore epithelial-specific protein expression such as E-cadherin [40]. However, targeting ZEB1 and ZEB2 with shRNAs along with adding a ROCK inhibitor led to complete reduction of stress fibers and restoration of epithelial protein expression. Taken together, these data provide evidence that inhibition of the TGF-β pathway regulates the transcriptional expression of epithelial-specific genes via the ZEBs, while other factors such as the Rho kinases are essential to re-establish the epithelial cell structure (Figure 1).

In summary, treatment strategies targeting the TGF-β signaling pathway are attainable antifibrotic options in kidney disease. Specifically, macromolecules such as BMP-7 and HGF have shown promising results in preclinical studies. Our own studies provide evidence regarding which specific signaling proteins and transcription factors are involved in the EMT reversal process, thus providing potential therapeutic targets.

## List of abbreviations used

EMT: epithelial to mesenchymal transition; JNK: c-Jun NH-terminal kinase; MEK1: MAPK/extracellular signal-regulated kinase; mTEC-KO: murine tubular epithelial cells from TGF-β knockout mouse; p38 MAPK: p38 mitogen-activated protein kinase; NMuMG: Namru murine mammary gland; ROCK: Rho kinase; TGF-β: Transforming Growth Factor β; TβRI: Transforming Growth Factor-β Receptor Type I.

## Competing interests

The authors declare that they have no competing interests.

## Authors' contributions

SD prepared the manuscript. FMH and JEM edited the manuscript. All authors read and approved the final manuscript. BNB, FMH, and JEM provided funding.
